# Leader Boundary-Spanning Behavior and Employee Voice Behavior: The Job Demands–Resources Perspective

**DOI:** 10.3390/bs13020146

**Published:** 2023-02-09

**Authors:** Jihye Lee, Dongwon Choi, Minyoung Cheong

**Affiliations:** 1 College of Transdisciplinary Studies, Daegu Gyeongbuk Institute of Science and Technology (DGIST), Daegu 42988, Republic of Korea; 2 Ewha School of Business, Ewha Womans University, Seoul 03760, Republic of Korea; 3 Department of Business Administration, School of Management, Kyung Hee University, Seoul 02447, Republic of Korea

**Keywords:** leader boundary-spanning behavior, self-efficacy, voice behavior, abusive supervision

## Abstract

Drawing on the job demands–resources model, we suggest and test a motivational mechanism that underlies the relationship between leader boundary-spanning behavior and employee voice behavior. Based on the field survey data of 383 leader-employee pairs collected from various organizations in South Korea, the results of our mediation model showed that leader boundary-spanning behavior, as a potential job resource, enhances employee voice behavior by increasing employee self-efficacy. The results of our moderated mediation model also showed that the focal leader’s abusive supervision, as a potential job demand, could attenuate the beneficial effect of leader boundary-spanning behavior on employee voice behavior by diminishing employee self-efficacy. These findings highlight the importance of leader boundary-spanning behavior in enhancing employee voice behavior, the roles of employee self-efficacy as a key mediating mechanism, and the focal leader’s abusive supervision as a preventable boundary condition within these relationships. Theoretical and practical implications are discussed.

## 1. Introduction

In volatile, uncertain, complex, and ambiguous (VUCA) organizational environments, one of the leaders’ critical goals is to sustain and expand their competitive advantages inside and outside the organizational boundaries [1]. To fulfill this goal, as much as leaders’ general inputs are crucial, employees’ practical inputs and proactive information sharing, by voicing up to their leaders, are essential [2,3]. This can be reflected in the form of employee voice behavior, which is generally viewed as a discretionary, prosocial, and challenging employee behavior [4,5].

Employees engage in voice behavior, such as expressing their thoughts and suggestions and sharing work-related knowledge, with the intent being the pursuit of desirable organizational improvements and/or changes [4]. Despite its potential benefits, employees often hesitate to engage in voice behavior, as this could involve demanding and even risky actions requiring careful resource consumption and allocation [6,7]. Furthermore, due to its discretionary characteristics, employees may decide not to speak up as they fear resource shortage [8]. Accordingly, scholars have identified an imperative role of leaders in providing sufficient resources and improved work conditions to enhance employee voice behavior.

Among the many forms in which leaders inject information, resources, and knowledge into their organizations, a special interest in leader boundary-spanning behavior is growing, as recent environmental changes emphasize leaders’ role in balancing internal and external activities [9,10,11,12]. Recent organization designs such as virtual organization, network organization, and team structure break down external barriers and, at the same time, lower the fences of organizational walls. Thus, leaders are expected to spend more time coordinating and controlling external relations to seek their competitive advantages [13].

Since leaders are in an advantageous position to access formal and informal resource flows and, therefore, influence decisions and/or negotiations with exclusive knowledge [14,15], previous studies showed leader boundary-spanning behavior have informational benefits across organizations [14,16,17,18]. In a similar vein, Yukl [19] demonstrated leader boundary-spanning behavior as one of four essential categories of effective leadership: task-oriented, relations-oriented, change-oriented, and external behavior. Despite its potential theoretical and practical contributions to leadership literature, the verification of how leader boundary-spanning behavior affects employee attitude and behavior remains overlooked [20]. Thus, to fill this research gap and identify its influences on various aspects of organizations, the current study examines the positive impact of leader boundary-spanning behavior and how it affects employees’ discretionary, prosocial, and challenging behavior: employee voice behavior.

We specifically draw on the job demands–resources (JD-R) perspective [21] to emphasize the impact of external resources and the informational benefits of leader boundary-spanning behavior on individual employee voice behavior. The JD-R model explains every job possesses demands and resources aspects. While job demands negatively influence employee work engagement and well-being, job resources positively impact those outcomes [21,22,23]. By igniting employees’ energy and making them feel supported by their organizations, job resources facilitate employees’ motivational process, resulting in positive work-related outcomes [24]. Recent meta-analysis showed that the JD-R model is increasingly used to linked to leadership; in particular, the most used connection is conceptualizing leadership as a job resource and/or demand itself [25]. Building on the insight from the JD-R model, we assert that leaders’ provision of external information/knowledge/resources to employees can be considered job resources on employee voice behavior. The current study also explores the psychological mechanism by which leader boundary-spanning behavior affects employee voice. Employees who interact with boundary-spanning leaders are likely to perceive resource gain and increased beliefs about how much control they have over their work environment (i.e., self-efficacy). Because engaging in voice requires additional resources [6,7], employees who perceive increased job/personal resources are likely to engage in a gain spiral, motivating to speak out and voice up. Thus, based on the JD-R model, we examine the mediating role of employee self-efficacy as a key mechanism linking leader boundary-spanning behavior to employee voice behavior.

Moreover, we posit that the focal leader’s abusive supervision would moderate the suggested motivational mechanism of leader boundary-spanning behavior on employee voice behavior through affecting employee self-efficacy. Previous studies noted that the positive effect of job resources on employees’ motivational process might be diminished in the presence of job demands, as it requires physical and psychological costs [24]. Among the various demanding work environments, we paid special attention to abusive supervision since it has been considered one of the representative job demands created by leaders [8,26,27,28]. Scholars have noted that supervisory abuse threatens employees’ resources by inducing the management with unfair and aggressive treatment [28,29]. Even though employees are equipped with sufficient information/knowledge/resources provided by their leader, they may lose their motivation to utilize those resources when they experience high levels of abusive supervision, thereby decreasing its influence on self-efficacy. Employees who perceive abusive supervision may try to avoid further resource loss by withholding their discretionary behaviors, such as voice behavior [30]. Therefore, using a moderated mediation framework, we suggest that the mediating effect of employee self-efficacy between leader boundary-spanning behavior and voice behavior varies depending on abusive supervision. As noted, abusive supervision is known to -detrimental factor through hostile treatments, and thus, the positive effect of leader boundary-spanning behavior is likely to weaken when the level of abusive supervision is high.

**Figure 1:**
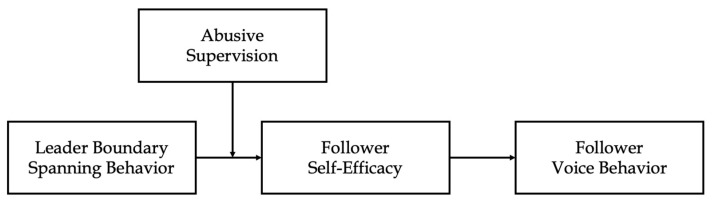
Hypothesized Research Model.

Overall, we integrate JD-R perspective with leader boundary-spanning behavior to accomplish below three research objectives. First, we suggest leader boundary-spanning behavior as a job resource and investigate its influence on employee discretionary behavior at the individual level. Specifically, we contribute to the leadership literature by emphasizing the critical role of leader boundary-spanning behavior following the increased attention on external activities in the business environment. Second, the current study examines the under-investigated psychological mechanism of leader boundary-spanning behavior as a job resource that can, directly and indirectly, influence employee voice behavior through increased employee self-efficacy. Third, this study investigates the boundary condition of leader boundary-spanning behavior (i.e., abusive supervision) and examines how this factor may limit the informational benefits of leader boundary spanning behavior on employees’ psychological and behavioral outcomes. By so doing, we try to fill research gaps in the respective literature by highlighting the importance of examining leader boundary-spanning behavior and its influences on various employee attitudes and behaviors and its potential boundary condition, thus contributing to the field of leader boundary-spanning behavior [10,11,12,19,20,31,32]. The overall research model of the current study is provided in Figure 1.

## 2. Theoretical Background and Hypotheses Development

### 2.1. Job Demands–Resources Model

Rooted in the job demand-control model [33], the job demands–resources model emphasizes each distinguished role of job demands and job resources as well as its interactive role in influencing employees’ work stress and motivation [22,24]. Job demands require psychological and physical burdens from the person in charge, such as workloads, time pressure, and physical environment difficulties [21,22]. Related studies of job demands mainly focused on its negative sides and investigated their effects on employees’ psychological costs, such as burnout, motivation, job enthusiasm, and job performance [23,24]. Job resources are known as the core of the motivational process, and are used to effectively achieve employees’ work goals (i.e., job control, development opportunities, and participation in decision making). Previous studies showed that job resources stimulate employees’ growth and development, and they also reduce job demands accompanying physical and psychological costs [23,24].

In workplaces, leaders play an important role in assigning work, coordinating the work processes, and providing the necessary resources to perform the respective tasks effectively. To date, some scholars have used the JD-R model to investigate how leadership affects employees’ attitudes and behaviors, and a recent meta-analysis has provided a systematic understanding of how leadership and JD-R theory can be connected [25]. According to the JD-R model, leaders’ boundary-spanning behavior can be considered as psychological, social, and organizational job resources. Employees would think the external information/resources/knowledge from outside the work group provided by their leaders as instrumental support [12,17]. The shared information and leveraged resources then generate personal job resources, such as new ideas created by the inflow and dissemination of external perspectives, which could enhance employees’ personal development and their performance [18,34]. The previous study showed that leader boundary-spanning behavior made employees perceive that they are receiving the required assistance to carry out their work, thus heightening their interests and job satisfaction [34,35,36]. Based on this JD-R model and the related empirical results, in the current study, we specifically focus on the motivating and knowledge-disseminating role of leader boundary-spanning behaviors and consider them as job resources, which can enhance employees’ voice behavior.

### 2.2. Leader Boundary-Spanning Behavior and Employee Voice Behavior

Boundary-spanning behavior refers to the actions that build and manage external parties by exploring and obtaining the resources and information through within an organization or across organizational boundaries [36,37]. Previous studies of boundary-spanning behavior have overlooked its complex dynamics and mainly focused on the team level phenomena by examining team boundary spanning or the positive effects of individual boundary-spanning behavior [32]. However, given that leaders have legitimate authority and unique network position to acquire and manage necessary resources from inside and outside of their groups, researchers in this realm have emphasized the role of leaders to facilitate interaction and information exchange within and across group boundaries [31,38]. Indeed, leader boundary-spanning behavior has been spotlighted as a way of effectively manage the flow of resources, information, and knowledge across external boundaries [9,11,17]. Previous studies showed that leader boundary-spanning behavior have informational benefits [14,15,16,17,18], as leaders occupy an advantageous position to access formal and informal resource flows and to influence decision or negotiation with exclusive knowledge [14,15].

Leader boundary-spanning behavior has been assorted several sub-dimensions. Ernst and Yip [39] proposed four ways to manage the social boundaries of work groups within the organization, including suspending, reframing, nesting, and weaving. Salem and colleagues [13] noted that leader boundary-spanning behavior comprises a broad set of externally oriented activities: acquiring resources and information from external parties in response to employees’ demands, building relationship with in- and out-stakeholders, and persuading outsiders to provide support. Recently, Marrone and colleagues [11] divided the leader boundary-spanning behavior into two dimensions: boundary loosening and boundary tightening. These classifications share some common ground in the inflow and dissemination of necessary external information, collaboration with key stakeholders, and interactions with other parties are essential activities of leader boundary spanning. Despite its effectiveness and versatility, few studies scrutinized the diverse perspectives of leader boundary-spanning behavior [20]. In particular, there is a lack of empirical studies investigating the potential mechanisms between leader boundary-spanning behavior and employee psychological and behavioral outcomes and its boundary conditions [40]. To fill this research gap, this study integrates the JD-R perspective with leader boundary-spanning behavior and examines why and how leader boundary-spanning behavior, as job resources, influences employees’ voice behavior.

Voice behavior refers to the voluntary communication of ideas, opinions, and concerns to improve organizational effectiveness [41,42]. Employees’ voice behavior, providing ideas and thoughts regarding various work-related issues, is crucial for continuous improvement and effective organizational decision making [5,42,43]. Despite the benefits, employees are often reluctant to voice up and across, as this demands higher resource consumption and potential risks such as damaging actors’ credibility and receiving a negative performance evaluation [6,44].

Exercising voice behavior depletes one’s resources because it involves maintaining attention to work-related affairs, identifying problems, and providing innovative ideas and suggestions [7,8,45]. Morrison [4] noted that voicing up is not necessarily the default option for employees. Employees may not engage in voice behavior when they perceive a resource shortage and face unfavorable conditions to speak up. Accordingly, scholars have examined those conditions and identified leadership as one of the important factors influencing employees’ intention to engage in voice behavior [38]. For example, employees increase their voice behavior when they have a positive and supportive relationship with their leader [46,47] and perceive their leader as a transformational or an ethical leader [48,49]. Further, employees engage in more voice behavior if they perceive their leader genuinely solicits their opinions by showing receptivity to the suggested ideas and thoughts [2,47]. In sum, the key to engaging more employee voice behavior is for leaders to provide adequate resources and improve work environments.

In line with this, we suggest leader boundary-spanning behavior could elicit employees’ voice behavior. Leaders who engage in boundary-spanning behaviors can deliver insightful information and knowledge to their organization, providing employees with ample work opportunities to access external resources and decreasing employees’ perception of resource loss [16]. Given that leader boundary-spanning behavior can manage the flow of job-relevant information and resources [18,34,35], focal employees might think of it as useful and valuable support from their leader. Employees working in such a supportive setting may decide to perform prosocial behavior in the way of reciprocity due to personal obligations [50,51]. As a result, employees with leaders who exhibit boundary-spanning behaviors are likely to engage in a higher level of voice behavior. Therefore, the following hypothesis is proposed:

**Hypothesis 1.**  *Leader boundary-spanning behavior is positively related to employee voice behavior*.

### 2.3. The Mediating Role of Employee Self-efficacy

Self-efficacy refers to individuals’ belief in their overall task-related capability [52]. Highly efficacious individuals set higher goals, initiate actions, and invest more effort and time in pursuing challenging goals even when they face obstacles [53]. As an imperative antecedent, previous research examined that the specific leader behaviors can exert significant influence on employees’ self-efficacy [54,55,56,57]. In the current study, we suggest leader boundary-spanning behavior could encourage employee voice behavior by increasing their self-efficacy.

As noted earlier, voice behavior could bring potential risks to employees as it challenges their status quo [3]. Additionally, it requires job and personal resources to facilitate since it needs sustained attention on work issues, creating fresh ideas, and delivering suggestions [8]. Because employee voice is discretionary in nature, employees would only be willing to engage in voice behavior if they have the assurance of benefits from speaking up and across [44]. With increased access to helpful resources from varied areas, employees working with boundary-spanning leaders are likely to cultivate personal control and agency. Scholars suggested that benefits from active interactions among individuals with different backgrounds enable the accumulation of diverse knowledge and viewpoints, which may increase individuals’ competence beliefs to achieve task goals successfully [58]. Boundary-spanning leaders bridge - employees and organizations to the external environments and motivate them to mobilize external players’ support and resources [14,35]. The literature on the JD-R model supports that leadership may impact employees’ job/personal resources which refers to one’s beliefs about how much control they have over their environment: self-efficacy [24]. Therefore, employees working with boundary-spanning leaders are likely to cultivate a ‘*can do*’ mindset and believe in their higher ability to deal with complex and demanding tasks.

In this regard, we expect that increased employees’ level of self-efficacy through leader boundary-spanning behavior encourages them to further engage in voice behavior. First, employees with increased self-efficacy would be equipped with high levels of task knowledge and creativity in problem solving [52]. These employees are more likely to voice their ideas on task-related issues as they believe their opinions would be respected and recognized by leaders and their peers. Walumbwa and colleagues [59] found that highly self-efficacious employees believe their leaders would be more receptive to their input. Second, increased self-efficacy could lead to employees dealing with their work stress more efficiently [60]. Voice behavior requires employees to clearly understand task-related and organizational issues/concerns clearly. It also pressures employees to communicate effectively with their leaders and coworkers [45,46], demanding much of their cognitive and emotional resources. Thirdly, according to Hobfoll [61], individuals with greater access to resources are less prone to losing and willing to invest and/or gain resources. Employees with high self-efficacy, which is considered as a valuable psychological resource [62], tend to take on new and challenging tasks and put up with difficulties to fulfill their goals [63,64]. This gain spiral of resources could lead employees to engage in more discretionary behaviors, such as employee voice behavior.

The literature on voice behavior has observed how resources provided by leader behaviors influence employee self-efficacy and play a vital role in enhancing employee voice behavior [65]. For example, Wang et al. [66] found that self-efficacy mediates the relations between ethical and paternalistic leadership and voice behavior. Li and coworkers [67] showed that self-efficacy and psychological safety mediate the effect of paradoxical leadership on employee voice. These findings suggest that employees with increased self-efficacy seek to resource gain by engaging in more discretionary behavior such as voice behaviors. Overall, by integrating the proposed relationships, we expect that leader boundary-spanning behavior may indirectly influence employee voice behavior by increasing employee self-efficacy. Thus, we propose:

**Hypothesis 2.**  *Employee self-efficacy mediates the positive relationship between leader boundary-spanning behavior and employee voice behavior*.

### 2.4. The Moderating Role of Abusive Supervision

Even though leader boundary-spanning behavior is helpful to encourage employees’ voice behavior by cultivating their sense of efficacy, the respective benefits of boundary management would be ineffective unless the leaders perform their internal roles successfully. To maximize the synergistic interactions of internal and external activities, leaders are responsible for properly managing both internal interactions with employees and external linkages with outsiders. Previous studies on boundary-spanning role of leaders noted the necessity of supportive internal process to make boundary-spanning behavior more effective [19,68]. Hence, the current study investigates the specific internally oriented leader behavior which could diminish the effect of leader boundary-spanning behavior on employee self-efficacy. Specifically, we suggest abusive supervision as an unfavorable internally oriented leader behavior that diminishes the positive relationship between leader boundary-spanning behavior and employee outcomes.

Abusive supervision refers to employees’ subjective perceptions of the extent to which their leaders engage in a sustained display of hostile, verbal, and non-verbal behaviors, excluding physical contact [27,69,70,71]. Abusive supervision has been studied as a powerful factor in understanding employees’ stressful situations that require sustained effort [22,24]. From the viewpoint of the JD-R model, leaders who exhibit abusive supervision can be viewed as representing the social and organizational aspects of job demands, which could deplete employees’ efforts at a high psychological cost. In the context of high levels of abusive supervision, employees should sustain their efforts to properly cope with physical and psychological costs. As such, abusive supervision has been considered as a representative job demand in the JD-R model [8,72]. Previous findings indicate that abusive supervision is associated with a wide variety of negative employee behaviors [69,70,71,73]. The harmful effects of perceived abusive supervision on employees’ affective and behavioral outcomes are well examined and provide sufficient evidence to consider it as a job demand.

The recent research of the JD-R model suggests that leadership can moderate the associations between job/personal resources and employees’ motivation processes [25]. In line with this, we assert that abusive supervision, as a job demand, can reduce the positive impact of job resources. Specifically, leaders’ abusive supervision could decrease employees’ self-efficacy by forcing them to utilize new external work resources. Employees’ psychological and motivational grit should be supported to effectively use the new work resources acquired through leader boundary-spanning behavior. From a motivational standpoint, however, abusive supervision would inhibit employees’ motivational processes as they diminish employees’ sense of self-determination. In other words, employees are less inclined to invest their cognitive, emotional, and psychological energy into their performance when they face motivational hurdles created by their leaders [73,74]. Therefore, abusive supervision undermines the resourceful effects of leader boundary-spanning behavior on both psychological and behavioral outcomes of employees. Hostile leaders’ constructive external role, such as boundary-spanning behavior, may bring more significant resource depletion of employees, since they experience both cognitive and emotional dissonance arising from the conflicting behaviors of their leaders. In this context, employees may not be able to accept and take advantage of an influx of resources their leaders provide. As a result, despite the benefits of leaders’ boundary spanning, employees feel less efficacious in exercising influence over the task environment to improvement or to helping others if the same leader engages in abusive supervision.

In addition, abusive supervision represents a chronic stressor threatening valued resources [74]. Leader abuse has been noted that threatens employees’ valued resources such as employment security and advancement opportunities [28,70]. Abuse supervision also causes a loss of personal resources (e.g., time and energy) because the target employees need to address or cope with leaders’ unfair and aggressive treatment [29,73]. Thus, victimized employees experience the depletion of personal resources necessary for improving their self-efficacy. Since control over their job is an essential resource available to employees [75], employees may not decide to exhibit discretionary behavior to sustain their remained resources. Abusive supervision can discourage employees with a boundary-spanning leader to deploy delivered external resources and information. Thus, we argue that the positive effect of leader boundary-spanning behavior on employee voice behavior through their self-efficacy is likely to be weakened when employees experience a high level of abusive supervision (i.e., Hypothesis 3).

Along with the above logic and reasoning, we further try to test the potential moderated mediation effect [76], in which leader boundary-spanning behavior is indirectly related to employee voice behavior through their self-efficacy, wherein this indirect linkage could vary on the level of abusive supervision. Specifically, when abusive supervision is high, the strength of the positive indirect effect of leader boundary-spanning behavior on employee voice behavior through their self-efficacy becomes weak. Taken together, we predict the following:

**Hypothesis 3.**  *Abusive supervision moderates the positive relationship between leader boundary-spanning behavior on employee self-efficacy, such that the positive relationship becomes weak when abusive supervision is high rather than low*.

**Hypothesis 4.**  *Abusive supervision moderates the indirect relationship between leader boundary-spanning behavior and employee voice behavior through self-efficacy, such that the positive indirect relationship becomes weaker when abusive supervision is high rather than low*.

## 3. Methods

### 3.1. Participants and Procedure

To test the hypothesized model, we collected data from the employees of business organizations in various industries, including manufacturing, telecommunication, and service sectors. Before conducting the survey, we asked the coordinators from the MBA administration office of a major university in South Korea to publicize the survey. With the support of them, we contacted part-time MBA students whose were fulltime employees during weekdays working for organizations. We briefly informed them that the purpose of this study was to examine the work dynamics of leadership and followers’ behavior response in organizations and then recruited employees and their supervisors. Participation was voluntary, and students were given extra credit. We used a snowball sampling method, asking initial participants to share the invitation in their own network.

To minimize potential common method bias effects, we collected the survey data from multiple sources by preparing the pairs of different types of questionnaire which included one for the employee and the other for immediate leader [77]. Therefore, the participants were given a cover letter outlining the study, a questionnaire, and a stamped envelope preaddressed to the researchers. We also delivered the purpose and voluntary nature of the study, the procedure for completing the survey (e.g., the time required for survey completion, how to return answered questionnaire), and the consent form. Moreover, to ensure the quality of the survey, we assured all participants that their responses would remain confidential and requested that they complete it truthfully. As we hypothesized, to understand the individual employee response to their leader behavior in workplace, which is an inherently dyadic phenomenon, we followed a dyadic data collection strategy. We included a researcher-assigned code number to ensure confidentiality and match each employee’s and leader’s responses. One leader filled out a questionnaire for only one employee (and vice versa). In order to incentivize respondents to complete the survey, we offered a small gift to each participant. Respondents returned their completed questionnaires directly to the researchers.

The questionnaires were initially distributed to 450 pairs, and a total of 392 pairs were obtained, indicating a response rate of 87.1%. From these, 5 pairs of questionnaires with mismatched dyads, 3 pairs with missing values of study variables, and 1 pair with unfaithful responses were excluded, and then 383 pairs were finally used for the study. For employees, 76% were male, and their average age was 35.44 years (SD = 5.49). For leaders, 81% were male and the average age was 43.40 years (SD = 5.18). The majority of employees and leaders held bachelor’s or higher degree.

### 3.2. Measures

All English language scales used in this study were translated into Korean using Brislin’s [78] conventional method of back translation. To assuage the concerns for same-source bias, leaders rated employee voice behavior, whereas employees rated their perception of leader boundary-spanning behavior, abusive supervision as well as their own self-efficacy. All items were measured on a seven-point Likert-type scale, ranging from strongly disagree (1) to strongly agree (7) except for the demographic data.

*Leader boundary-spanning behavior*. Leader boundary-spanning behavior was measured with 6 items developed by Marrone et al. [36]. We changed the subject from “this employee” to “my supervisor” in order to ask employees to rate the level of their leader’s boundary-spanning behaviors. A sample item read, “My supervisor acquires resources and access (e.g., access to information) for the team.” (α = 93).

*Self-efficacy*. Self-efficacy was assessed with an eight-item scale developed by Chen et al. [52]. A sample item was, “I am confident that I can perform effectively on many different tasks.” (α = 94).

*Abusive supervision*. To assess the perception of abusive supervision, we used a shortened five-item version of Tepper [27,79]. A sample item of abusive supervision is “My immediate supervisor makes negative comments about me to others.” (α = 93).

*Voice behavior*. Leader assessed their employee’s voice behaviors using a six-item scale taken from Van Dyne and LePine [5]. An example item of voice behavior is, “This employee speaks up and encourages others in the group to get involved in issues that affect the group.” (α = 92).

*Control variables*. Boundary-spanning activities in general and leader boundary-spanning behavior in particular entails characteristics of workforce diversity, which encourage distinct perceptions and behaviors among different gender and ages of employees [12]. Thus, we controlled employees’ demographic variables such as gender, age, and education level, which have been statistically controlled in existing studies examining the relationships between leader boundary-spanning behavior and employees’ behavioral outcomes [32].

## 4. Results

### 4.1. Preliminary Analysis

We conducted a confirmatory factor analysis to verify the construct validity of the hypothesized variables. The results showed that the hypothesized four-factor model fit well to the data (χ^2^ (269) = 995.18, CFI = 0.91, TLI = 0.90, and RMSEA = 0.08). Additionally, we compared the hypothesized model with alternative models. As presented in Table 1, the results indicated that the hypothesized model is superior to all other alternative models.

### 4.2. Analytic Strategy

To test our hypotheses, we conducted hierarchical regression analysis using SPSS 22.0. Table 2 presents the information for descriptive statistics and correlations among the study variables.

**Figure 2:**
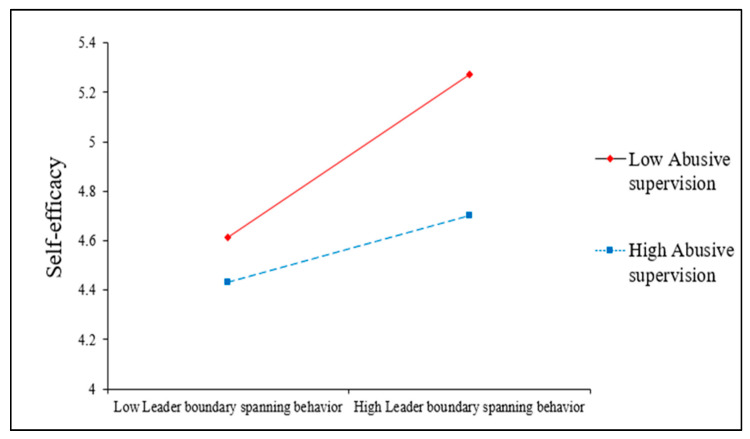
Moderating Effect of Abusive Supervision in the Relationship between Leader Boundary-Spanning Behavior and Self-Efficacy.

As shown in Table 3, leader boundary-spanning behavior had a positive effect on employees’ voice behavior (*b* = 0.10, *p* < 0.05), thereby supporting Hypothesis 1. Hypothesis 2 proposed that self-efficacy mediates the relationship between leader boundary-spanning behavior and employee voice behavior. We test the proposed mediation effects by using both hierarchical regression and bootstrapping approaches. As predicted, the all requirements of mediation are satisfied (see Table 3), and bootstrapping results confirmed the mediating role of self-efficacy (95% bias-corrected Cis from 0.02 to 0.09, excluding zero in the CI); thus, Hypothesis 2 was supported. The results shown in Table 4 demonstrated that the interaction term of leader boundary-spanning behavior and abusive supervision on self-efficacy was significant (*b* = −0.08, *p* < 0.01). In support of Hypotheses 3, Figure 2 graphically depicts the moderating effects of abusive supervision on the relationship between leader boundary-spanning behavior and employees’ self-efficacy.

Finally, Hypothesis 4 proposed that the indirect effect of employee self-efficacy between leader boundary-spanning behavior and employee voice behavior would be changed by the level of abusive supervision. To test conditional indirect effect, we followed a procedure recommended by Preacher and colleagues [76] using SPSS macro. As predicted, the indirect effect of between leader boundary-spanning behavior on voice behavior via self-efficacy was significant when abusive supervision was low. On the other hand, under high levels of abusive supervision, the same indirect effect was also significant but weaker (see Table 5). Thus, Hypothesis 4 was supported.

## 5. Discussion

### 5.1. Theoretical Implications

Despite the importance of discretionary actions of employees at work and the substantial role of leadership in facilitating self-initiative actions of employees at work [19], previous research in this domain falls short of explaining whether and how leader boundary-spanning behavior affects employee voice behavior. Building on the notion of JD-R model [21], the current study specified a mechanism (i.e., employee self-efficacy) as well as a boundary condition (i.e., abusive supervision) in the relationship between leader boundary-spanning behavior and employee voice behavior.

We believe the current research contributes to the existing literature on leadership, boundary spanning, and voice behavior for the following ways. First, our findings contribute to the leadership literature by suggesting the need to investigate external leader role (i.e., leader boundary-spanning behaviors) in predicting employees’ proactive activities at the individual level. The extant research in leadership has mostly paid attention to internal leader roles despite the increasing significance of leaders’ roles in managing external boundaries [9,10,38]. Although previous studies examined the effectiveness of leader boundary-spanning behavior in the project team context [11,14,17], there remains a void in examining important individual-level work outcomes. The present study specifically articulated the positive relationship between leader boundary-spanning behavior and employee voice behavior, thus extending our understanding of the role of leaders’ boundary management, specifically at the individual level.

In addition, we extended leadership research by adopting the lens of JD-R to explain the effect of leader boundary-spanning behavior and that of abusive supervision. Applying the JD-R model, we revealed that leader boundary-spanning behavior plays the role of job resource, since it can help employees to link additional information and advice from within and across other groups, achieving their work goals. On the other hand, in keeping with previous research [26,49], we considered abusive supervision as a job demand, as dealing with these behaviors requires employees’ physical and psychological costs. Our study complements previous works by proposing different types of leader behaviors that can act as beneficial and/or detrimental to psychological processes, thus shaping employees’ motivation to engage in voice behavior.

We also offered a deeper understanding on leader boundary-spanning behavior by elaborating on a boundary condition that diminishes the positive impact of boundary spanning. Our study examined abusive supervision as a contingent factor that changes the positive effect of leader boundary-spanning behavior on employees’ psychological and behavioral outcomes. Previous studies have demonstrated abusive supervision’s negative influences on employee emotions, attitudes, and behaviors [70,71]. In line with this, our findings underscore the destructive impact of abusive leaders by showing that it could cancel out the constructive impact of boundary-spanning activities. This suggests that leaders should practice and perform both their internal and external roles effectively to make a synergetic relationship, thereby maximizing the positive leadership influences.

Finally, when it comes to the contribution to voice literature, our study suggested a novel antecedent of voice behavior: leader boundary-spanning behavior. We further addressed how leader boundary-spanning behavior relates to employee voice behavior by suggesting a psychological mechanism underlying the relationship. Our findings revealed that leader boundary-spanning behavior could help to cultivate employees’ sense of efficacy and to facilitate subsequent discretionary behavior by providing an extended set of knowledge, resources, and networks required to speak up. Based on the current finding, future research could delve into other psychological processes by which leader boundary spanning could influence employee voice behavior.

### 5.2. Practical Implications

In terms of practical implications, first, our findings suggest that merely facilitating leader boundary-spanning behavior is not enough; instead, organizations should prevent leaders from engaging in negative leader behaviors such as abusive supervision. Indeed, prior studies have noted that boundary-spanning activity is a taxing, demanding and challenging behavior [36,80]. As individuals perform boundary-spanning activities, they may experience role conflict and stress since it requires individual actors’ limited resources and competes against within-boundary activities [80,81]. Thus, it is important that HR managers and organizations design treatment programs to manage those leaders’ stress and cope with their role overload. For sure, HR managers could also consider hiring or training leaders with certain traits or capabilities that could facilitate exhibiting boundary-spanning behavior. For example, leaders who display signs of boundary-spanning self-efficacy, role ambiguity, and certain network characteristics (i.e., structural hole) are more likely to engage in boundary-spanning activity [15,36,40].

Second, this study also includes a practical message to facilitate voice behaviors in organizations. As mentioned, practitioners and researchers have acknowledged that organizations should rely on employees’ suggestions and concerns to handle organizational processes and crises [41,42]. Given that, our study reveals that only employees with plenty of job resources are able to engage in such discretionary actions. Thus, HR managers need to create a work environment by providing job resources in the way of instrumental as well as emotional support. Besides leader boundary-spanning behavior, organizations should consider other types of leader behavior or coworker influences that can offer job resources. For instance, coworker knowledge sharing can provide valuable job resources by sharing their expertise and experience, which invites employee voice behavior [7].

Third, in considering the detrimental impact of abusive supervision, organizations and HR managers may modify the selection procedures by checking whether candidates have certain traits linked to abuse. According to the previous finding that abusive individuals are rarely able to change their behavior, organizations may benefit from identifying traits that may lead to abuse early in the selection process and decrease the possibility that employees will engage in such destructive behaviors [82]. Additionally, organizations should provide leadership programs for all leaders to make a better self-management and improve their communication skills, and inculcate the understanding that abuse is preventable. For employees, organizations should create an environment to strengthen personal resources with their jobs and empower more flexibility in work situations, which allows employees to deal with various job demands more proactively. In general, employees do not have the power to eliminate the occurrence of abusive supervision; however, they have the power to change their reactions as more proactively trying to ameliorate the situation. In line with previous findings that employees’ proactive mobilizing job resources and work environment can mitigate the harmful effects of abusive supervision [26], organizations may provide guidance to develop their job-related skills and capabilities to better cope with abusive supervision. Furthermore, organizations may develop policies and procedures aimed at anonymously reporting abuse occurrences and curbing such behavior.

### 5.3. Limitations

The current study has several limitations. First, when it comes to research design, we cannot infer causality from our results, due to the cross-sectional study design. In a similar vein, although we collected data from two different sources to relieve potential common method bias, there still remains the concern for common method bias due to the self-report data in measuring study variables. Future research could be fruitful to replicate the current study’s findings by adopting longitudinal design or conducting experiments to verify the causal relationships of the suggested model.

Second, our study also reveals a theoretical limitation in examining a more comprehensive mechanisms and boundary conditions. While there might be many more possible mechanisms and boundary conditions, we only consider one mediator and one moderator in our study. Searching for the potential mediating/moderating variables within the associations will broaden our understanding of the leader boundary-spanning behavior. Based on our findings, future research could investigate more diverse sets of mechanisms as well as boundary conditions, thus achieving theoretical extensions. Such accumulation of individual studies will provide an opportunity for researchers to conduct a meta-analysis that allows a more comprehensive investigation.

Third, in terms of generalizability, there is a limitation in the context of the sample in the current study. We collected the survey data from the employees in one country (i.e., South Korea); thus, our results could be influenced by the cultural context of South Korea. It may limit the generalizability of our findings to organizations with different cultural contexts. Accordingly, we recommend future research to take more culturally diverse samples so that we can examine whether our findings can be replicated and generalized.

### 5.4. Conclusions

In the face of the organizational context that requires employees’ initiative actions, this study highlights the role of the leader in facilitating employee voice behavior; specifically, we suggest that not only internal management activity, but also external management activity (i.e., leader boundary-spanning behavior) is important for leaders. By adopting theoretical lens of JD-R model, we further specified a psychological mechanism (i.e., employee self-efficacy) and a boundary condition (i.e., abusive supervision) in the relationship between leader boundary-spanning behavior and employee voice behavior. Despite the above limitations, we believe this research makes a unique contribution to the existing literature on leadership, voice, and boundary spanning by examining an integrative moderated mediation model delineating how leader boundary-spanning behavior influences employee voice behavior. We hope our study could motivate future scholars to consider leader boundary-spanning behavior as an important facilitator of proactive behaviors at work; likewise, we also encourage future research expand theoretical framework by examining a more various mechanisms and boundary conditions, deepening our understanding on leader boundary-spanning behavior.

## Figures and Tables

**Table 1:** Confirmatory Factor Analysis.

Model	χ^2^	df	Δχ^2^	CFI	TLI	RMSEA
Four-factor model ^a^	995.18	269	-	0.91	0.90	0.08
Three-factor model ^b^	2572.57	272	1577.39 ***	0.72	0.70	0.14
Two-factor model ^c^	3930.44	274	2935.26 ***	0.56	0.52	0.18
One-factor model ^d^	5426.93	275	4431.75 ***	0.38	0.32	0.22

Note. CFI = Comparative Fit Index, TLI = Tucker–Lewis Index, RMSEA = root-mean-square error of approximation. ^a^ Four-factors: Leader boundary-spanning behavior; Employee’s self-efficacy; Abusive supervision; Voice behavior. ^b^ Three-factors: Leader boundary-spanning behavior; Employee’s self-efficacy and Abusive supervision combined; Voice behavior. ^c^ Two-factors: Leader boundary-spanning behavior, Employee’s self-efficacy, and Abusive supervision combined; Voice behavior. ^d^ One-factor: Leader boundary-spanning behavior, Employee’s self-efficacy, Abusive supervision, Voice behavior combined. *** *p* < 0.001 (two-tailed).

**Table 2:** Means, Standard Deviations, and Intercorrelations of Variables.

	Mean	S.D.	1	2	3	4	5	6	7
1. Gender ^a^	1.28	0.45							
2. Age ^a^	35.53	5.73	−0.37 ***						
3. Education ^a^	3.04	0.65	−0.11 *	0.08					
4. Leader Boundary-Spanning Behavior ^a^	5.31	1.08	0.01	−0.03	0.11 *	(0.93)			
5. Self-efficacy ^a^	5.19	0.77	−0.07	0.09	0.10	0.38 ***	(0.94)		
6. Abusive Supervision ^a^	2.02	1.09	0.02	0.02	−0.03	−0.57 ***	−0.32 ***	(0.93)	
7. Voice Behavior ^b^	4.77	0.84	−0.01	−0.01	0.18 ***	0.14 **	0.21 ***	−0.30 ***	(0.92)

Note. N = 383. Reliabilities are on the diagonal in parentheses. ^a^ These variables were measured from focal employees. ^b^ Leader rating. * *p* < 0.05; ** *p* < 0.01; *** *p* < 0.001 (two-tailed).

**Table 3:** Hierarchical Regression Results.

	Self-Efficacy		Voice Behavior		
	Model 1	Model 2	Model 3	Model 4	Model 5
**Step 1. Control Variables**					
Gender	−0.08	−0.08	0.06	0.06	0.07
Age	0.01	0.01	0.01	0.01	0.01
Education	0.12	0.07	0.23 ***	0.22 **	0.20 **
**Step 2. Main Effect**					
Leader Boundary-Spanning Behavior		0.27 ***		0.10 *	0.05
**Step 3. Mediator**					
Self-efficacy					0.18 **
Overall *F*	2.50	17.65 ***	4.72 **	5.15 ***	6.17 ***
*R* ^2^	0.02	0.16	0.04	0.05	0.08
Δ*F*		61.94 ***		6.23 *	9.80 *
Δ*R*^2^		0.14		0.02	0.02
Bootstrap Results for Indirect Effect					
		Effect	SE	LL 95%CI	UL 95% CI
Effect		0.05	0.02	0.02	0.09

Note. N = 383. Entries are unstandardized regression coefficients. * *p* < 0.05; ** *p* < 0.01; *** *p* < 0.001 (two-tailed). Bootstrap sample size = 10,000. LL = lower limit; CI = confidence interval; UL = upper limit.

**Table 4:** Hierarchical Regression Results for Moderated Mediation.

	Self-Efficacy				Voice Behavior				
	Model 1	Model 2	Model 3	Model 4	Model 5	Model 6	Model 7	Model 8	Model 9
**Step 1. Control Variables**									
Gender	−0.08	−0.08	−0.07	−0.06	0.06	0.06	0.08	0.09	0.09
Age	0.01	0.01	0.01	0.01	0.01	0.01	0.01	0.01	0.01
Education	0.12	0.07	0.07	0.08	0.23 ***	0.22 ***	0.23 ***	0.23 ***	0.23 ***
**Step 2. Main Effect**									
Leader Boundary-Spanning Behavior (LBSB)		0.27 ***	0.20 ***	0.22 **		0.10 *	−0.05	−0.04 ***	−0.07
**Step 3. Moderator**									
Abusive Supervision (AS)			−0.12 **	−0.19 **			−0.26 ***	−0.30 ***	−0.28 ***
**Step 4. Interaction**									
LBSB ∗ AS				−0.08 **				−0.05	−0.04
**Step 5. Mediator**									
Self-efficacy									0.13 *
Overall *F*	2.50	17.65 ***	16.20 ***	15.65 ***	4.72 **	5.15 ***	11.22 ***	9.91 ***	9.25 ***
*R* ^2^	0.02	0.16	0.17	0.20	0.04	0.05	0.13	0.14	0.15
Δ*F*		61.94 ***	8.93 **	10.83 **		6.23 *	33.73 ***	3.07	4.70 *
Δ*R*^2^		0.14	0.02	0.02		0.01	0.08	0.01	0.01

Note. N = 383. Entries are unstandardized regression coefficients. * *p* < 0.05; ** *p* < 0.01; *** *p* < 0.001 (two-tailed).

**Table 5:** Moderated Mediation Results.

Moderator	Level	Voice Behavior			
		Conditional Indirect Effect	SE	LL 95% CI	UL 95% CI
Abusive Supervision	Low	0.06	0.02	0.02	0.10
	High	0.02	0.01	0.01	0.05

Note. N = 383. Bootstrap sample size = 10,000. LL = lower limit; CI = confidence interval; UL = upper limit. Control variables: gender, age, and education level.

## Data Availability

The data presented in this study are available on request from the first author.
